# Supplementary material for: GABAergic signaling linked to autophagy enhances host protection against intracellular bacterial infections
Source: Nat Commun. 2018 Oct 10;9:4184. doi: 10.1038/s41467-018-06487-5 (PMC6180030; doi:10.1038/s41467-018-06487-5)
Supplement: Supplementary file 1 — Supplementary Information [file 41467_2018_6487_MOESM1_ESM.pdf]

Supplementary Information

GABAergic signaling linked to autophagy enhances host protection against intracellular bacterial infections

Kim *et al.*

Supplementary Figure 1

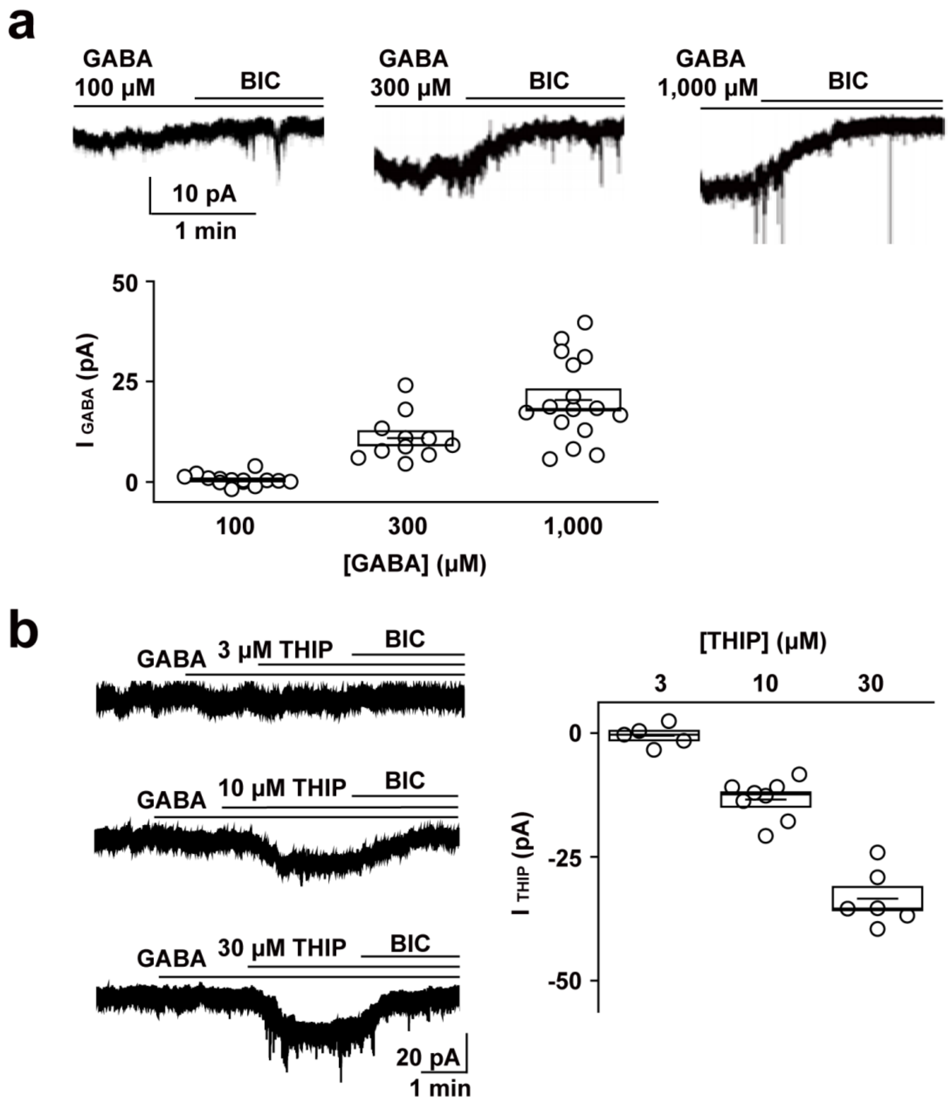

Supplementary Figure 1. Characterization of GABA-induced currents in BMDMs. (**a**) Representative traces showing tonic $GABA_AR$ current in the presence of GABA (upper). Tonic current amplitudes are summarized in box plot (*n* = 12 – 16, lower). Tonic $GABA_AR$ current was defined as the difference between the holding current before and after $GABA_AR$ antagonist, bicuculline (BIC, 50 μM, upper). Drugs were continuously perfused at a rate of ~3 ml/min with the extracellular recording solution. (**b**) Tonic $GABA_A$ currents generated by $GABA_AR$ δ subunit agonist, THIP in the presence of GABA (100 μM, left). THIP-induced

current amplitudes are summarized in box plot (n = 5 – 8, right). Traces are representative of fifteen independent experiments (**a**,**b**).

Supplementary Figure 2

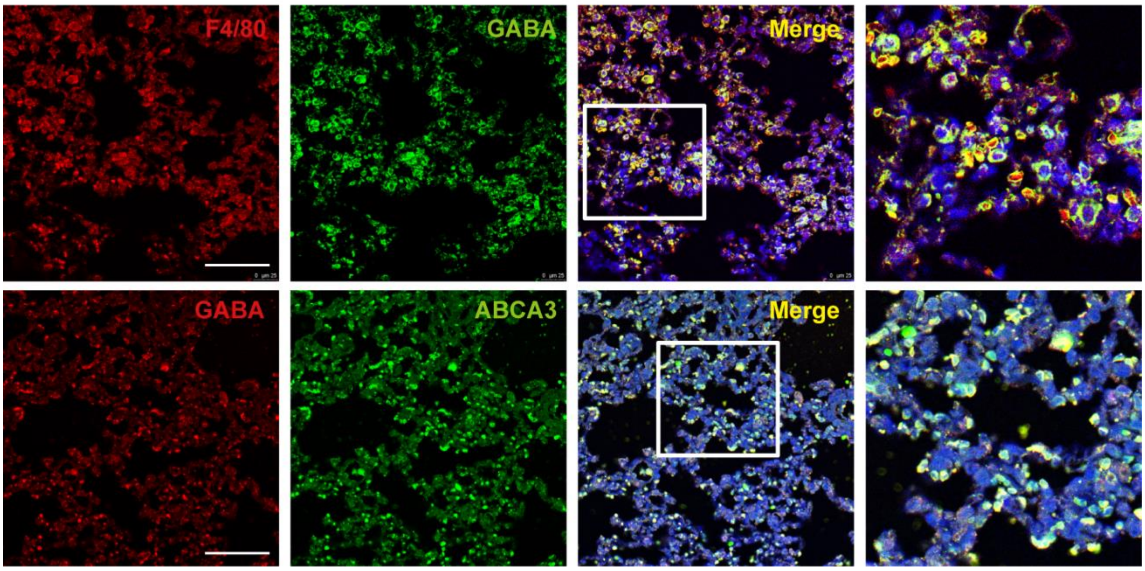

Supplementary Figure 2. Macrophages and AE II cells express GABA. GABA immunoreactivity in F4/80- or ABCA3- positive cells in lung tissues from uninfected mice. Blue color from DAPI for visualization of nuclei. Data are representative of three independent experiments. Scale bars, 25 µm.

Supplementary Figure 3

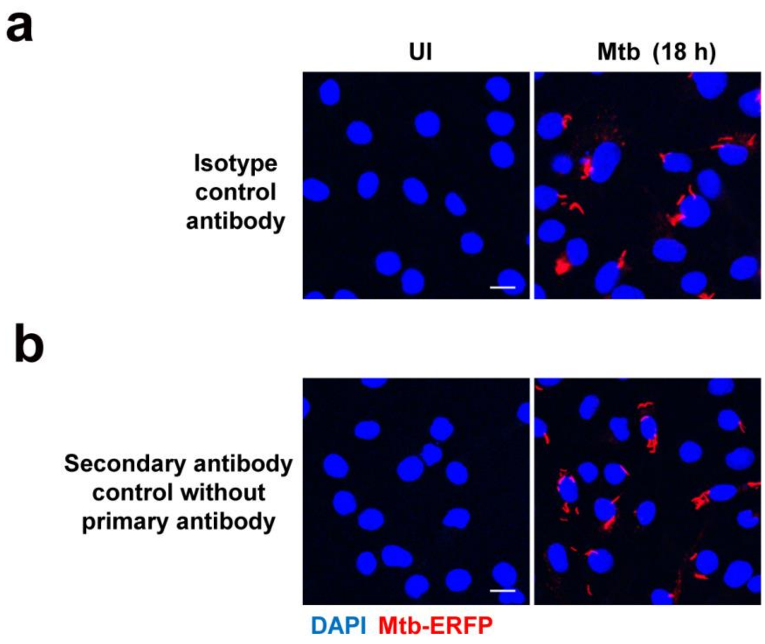

Supplementary Figure 3. Validation of specificity of primary and secondary antibodies for GABA staining. (**a** and **b**) BMDMs were infected with Mtb-ERFP (MOI of 5) for 18 h and then stained with rabbit isotype control (**a**) or no primary antibody negative control (**b**). Scale bars, 15 μm. UI, uninfected. Images are representative of three independent experiments (**a**,**b**).

Supplementary Figure 4

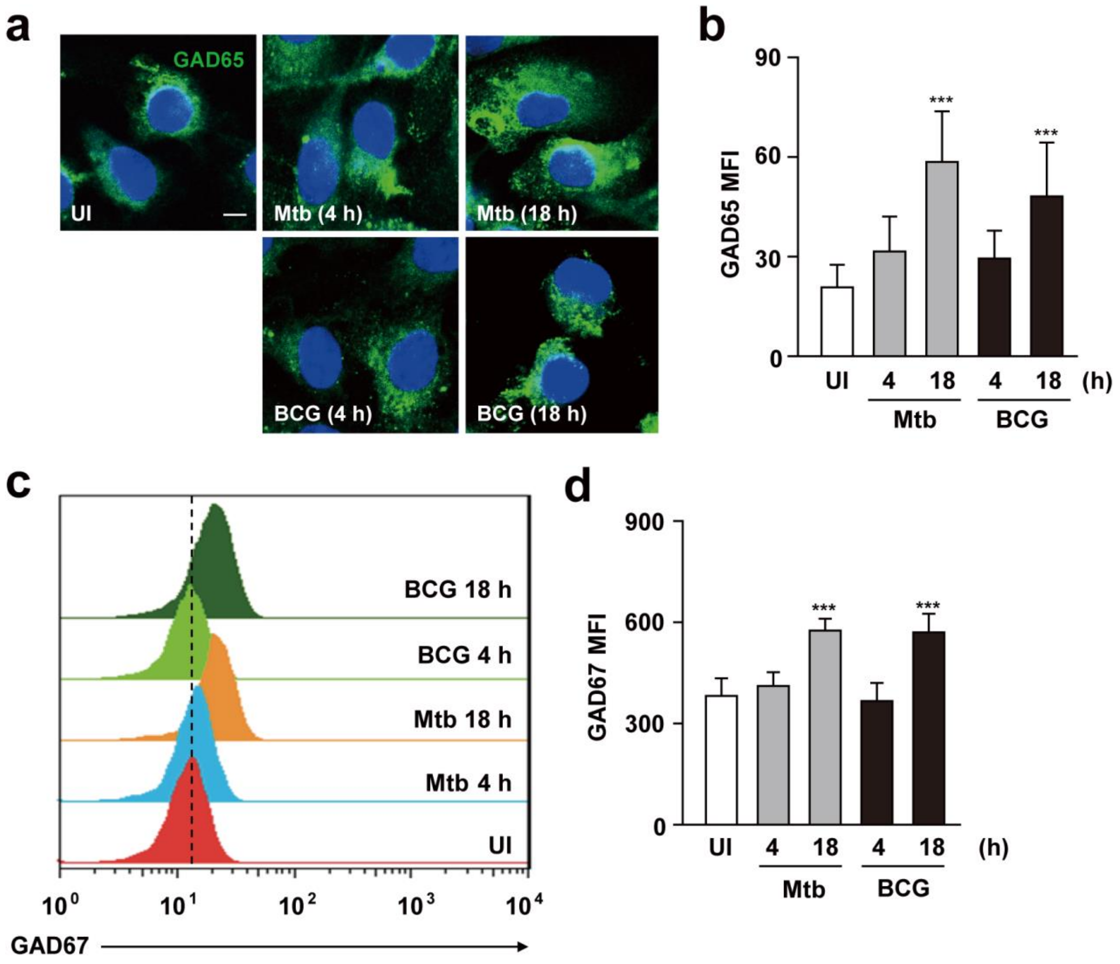

Supplementary Figure 4. Mycobacterial infection increases the expression levels of GAD65 and GAD67 in macrophages. (**a** and **b**) BMDMs were infected with Mtb (MOI of 5) or BCG (MOI of 5) for the indicated times and then stained with GAD65 (green) and DAPI (for nuclei; blue). (**a**) Cells were visualized by confocal microscopy. Scale bars, 7 µm. (**b**) Average MFIs of GAD65. (**c** and **d**) BMDMs were infected with Mtb (MOI of 5) or BCG (MOI of 5) for the indicated times. (**c**) The expression of GAD67 was analyzed by flow cytometry. Representative gating strategy is shown in Supplementary Fig. 5. (**d**) Average MFIs of GAD67. ***$p < 0.001$. UI, uninfected. Statistical significance was determined by one-way ANOVA (**b**,**d**). Data shown (means ± SEM) represent combined results of duplicate from two experiments (**b**,**d**). Data are representative of at least three independent experiments.

Supplementary Figure 5

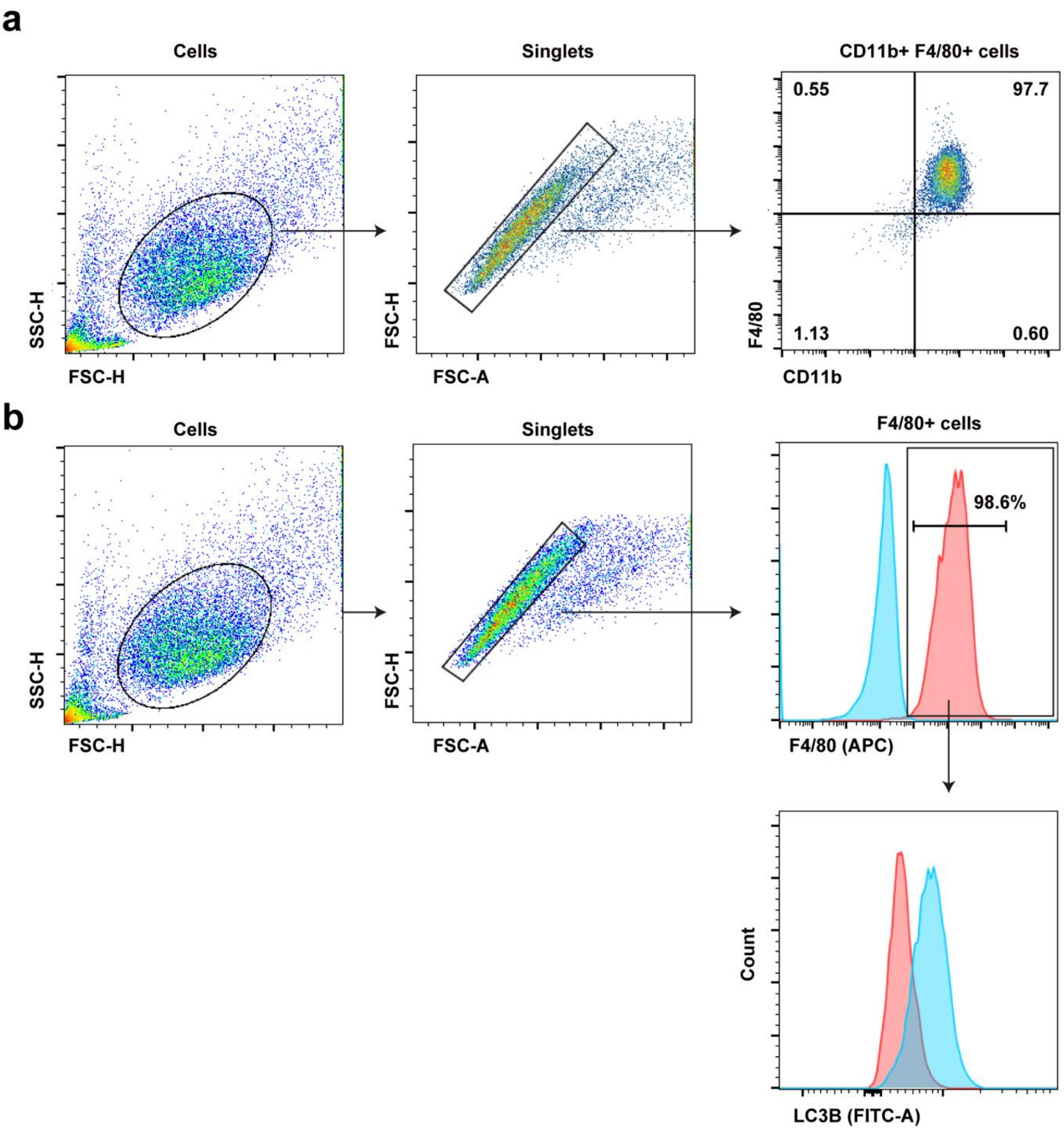

Supplementary Figure 5. FACS gating strategy. (**a**) Representative FACS analyses of BMDMs stained with CD11b and F4/80 surface markers confirming 97% CD11b+/F4/80+ BMDMs population. (**b**) After the exclusion of doublets and debris, the F4/80-positive gated population in samples was subjected to histogram analysis of LC3B or GAD65/67 expression (a representative image of FACS analysis; bottom).

Supplementary Figure 6

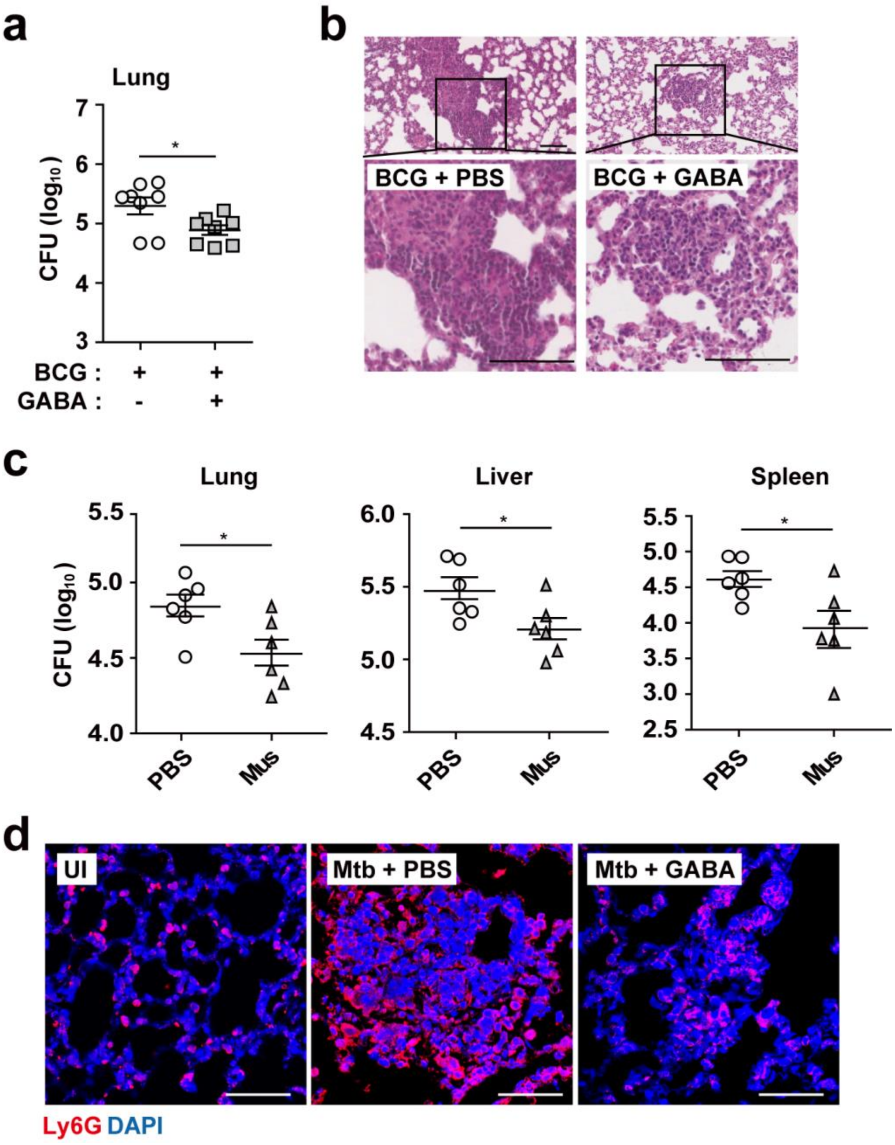

Supplementary Figure 6. GABAergic activation promotes antimicrobial responses against mycobacterial Infection *in vivo*. (**a**) Mice (*n* = 8 per group) were infected i.n. with BCG (2 x 10^{6} CFU) and treated with PBS or GABA (daily i.p. 200mg/kg), and monitored at 7 dpi. The bacterial loads in lung were determined by CFU assay. (**b**) Representative H&E-stained images of lung tissue of mice treated as in Fig. **2b**. Scale bars, 100 μm. (**c**) Mice (*n* = 6, per group) were infected i.v. with BCG (1 x 10^{7} CFU), treated with PBS or muscimol (Mus; daily i.p. 2 mg/kg), and monitored at 17 dpi. (**c**) The bacterial loads in lung, liver, and spleen

were determined by CFU assay. (**d**) Representative Ly6G imaging data from multiple lesions of lungs as in Fig. **2a.** Scale bars, 50 μm. *$p < 0.05$. UI, uninfected. Statistical significance was determined by Mann-Whitney U test (**a**, **c**). Data (means ± SEM) are pooled from three independent experiments. Images are representative of two (**a**,**c**) or three (**b,d**) independent experiments. Each symbols represents one animal (**a**,**c**).

Supplementary Figure 7

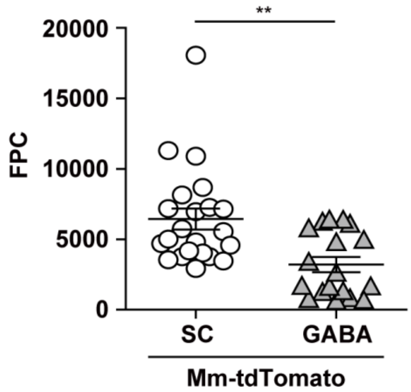

Supplementary Figure 7. GABA treatment enhances antimicrobial responses in zebrafish model. Zebrafish embryos were i.v. infected with *M. marinum*-tdTomato (200 CFU) and treated with vehicle (n = 22) or GABA (1 mM, n = 18). Bacterial loads (FPC; fluorescent pixel counts) were measured by fluorescence images at 4dpi. **$p < 0.01$. SC, solvent control (0.1% PBS in egg water). Statistical significance was determined by unpaired *t*-test. Data are representative of at least three independent experiments. Each symbol represents one animal.

Supplementary Figure 8

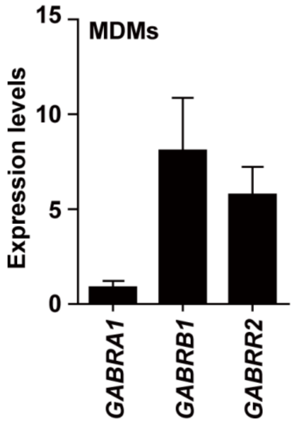

Supplementary Figure 8. Expression of GABA_AR subunits in human MDMs. In human MDMs, three GABA_AR subunit mRNAs were determined using the $\Delta\Delta$Ct method and normalized to the reference gene G*APDH*. Data represent the combined results of three independent experiments.

Supplementary Figure 9

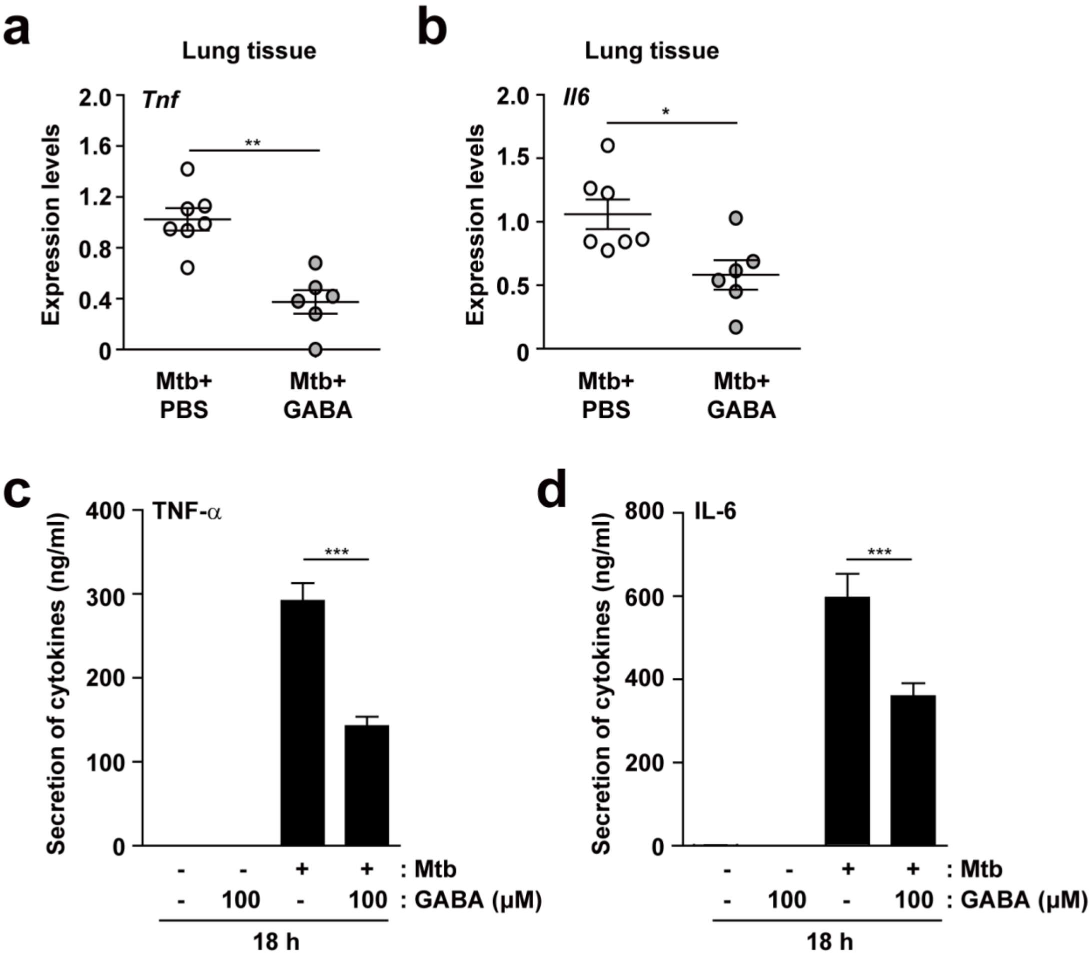

Supplementary Figure 9. GABA-mediated regulation of inflammatory cytokine generation in BMDMs and lung tissues during mycobacterial infection. (**a** and **b**) qRT-PCR analysis of *Tnf* and *Il6* mRNA levels in lung tissue of mice treated as in Fig. 2a. (**c** and **d**) BMDMs were infected with Mtb (MOI of 5), incubated with GABA (100 μM) for 18 h, and the expression of TNF-α and IL-6 proteins was analyzed by ELISA**.** *p < 0.05, **p < 0.01, and ***p < 0.001. ns, not significant. Statistical significance was determined by Mann-Whitney U test. Data shown (means ± SEM) represent combined results of duplicate from two experiments (**c**,**d**). Data are representative of at least three independent experiments. Each symbols represents one animal (**a**,**b**).

Supplementary Figure 10

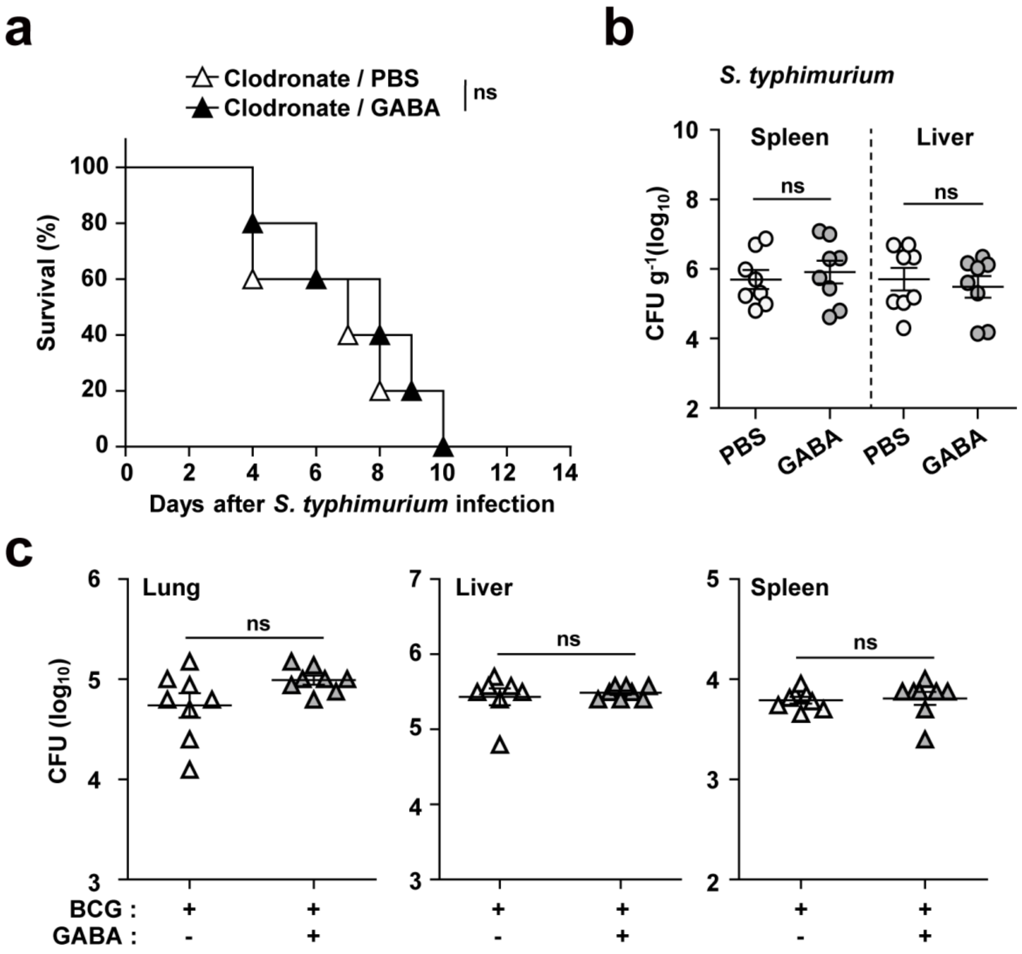

Supplementary Figure 10. Macrophage depletion abrogates the GABAergic host defense against salmonella and mycobacterial infection. (**a** and **b**) Mice were injected i.v. with clodronate liposomes (200 μl) 24 h before oral infection with *S. typhimurium* (1×10^8 CFU). After infection, PBS or GABA (200 mg/kg) were injected i.p. daily for 7 days. (**a**) Survival of mice (PBS; n = 5 or GABA; n = 6). (**b**) Viable cell count of intracellular *S. typhimurium* in spleen and liver of mice treated with PBS or GABA (n = 8 per group) at 5 dpi. (**c**) Mice were injected i.v. (200 μl) and i.n. (100 μl) with clodronate liposomes 24 h before BCG infection. Mice (n = 8 per group) were infected i.v. with BCG (1×10^7 CFU), treated with PBS or GABA (daily i.p. 200 mg/kg), and monitored at 7 dpi. The bacterial loads in lung, liver, and spleen were determined by CFU assay. ns, not significant. Statistical significance was

determined by Mann-Whitney U test (**b**,**c**) or log-rank (Mantel-Cox) test (**a**). Data (means ± SEM) are pooled from two independent experiments (**a**,**b**). Images are representative of two independent experiments (**c**). Each symbols represents one animal (**b**,**c**).

Supplementary Figure 11

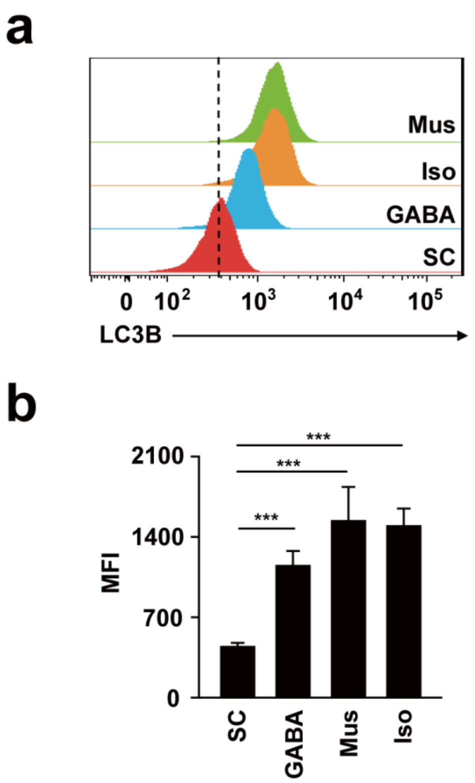

Supplementary Figure 11. GABAergic activation increases LC3B expression in macrophages. (**a** and **b**) BMDMs were incubated with GABA (100 μM), muscimol (Mus; 100 μM), or isoguvacine hydrochloride (Iso; 100 μM). (**a**) The expression of LC3B was analyzed by flow cytometry. Representative gating strategy is shown in Supplementary Fig. 5. (**b**) Average MFIs of LC3B. ***$p < 0.001$. SC, solvent control (0.1% PBS). Statistical significance was determined by one-way ANOVA (**b**). Data shown (means ± SEM) represent combined results of triplicate from two experiments (**b**). Data are representative of three independent experiments (**a**).

Supplementary Figure 12

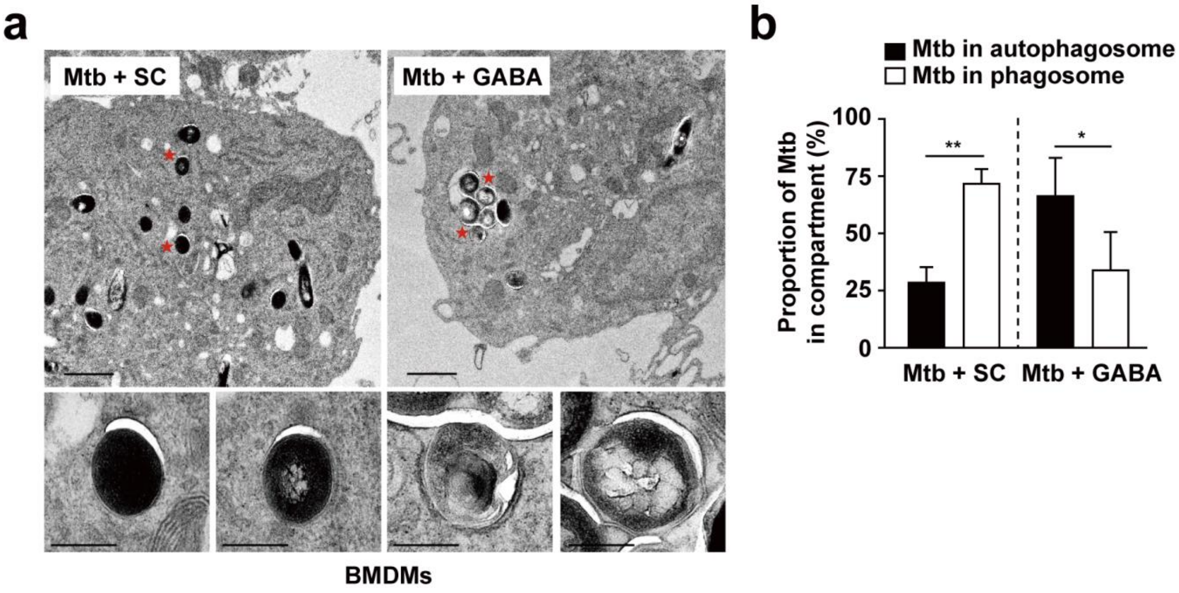

Supplementary Figure 12. Ultrastructural analysis of GABA-induced autophagic vesicles in macrophages during mycobacterial infection. (**a** and **b**) Representative low- (top) and high-magnification (bottom) transmission electron micrographs of BMDMs. Scale bars, 1 μm (top) and 200 nm (bottom). (**b**) Quantitative data of phagosomes and autophagosomes containing Mtb. *p < 0.05 and **p < 0.01**.** SC, solvent control (PBS). Statistical significance was determined by Mann-Whitney U test (**b**). Data shown (means ± SEM) represent combined results of triplicate from two experiments (**b**). Images are representative of two independent experiments (**a**).

Supplementary Figure 13

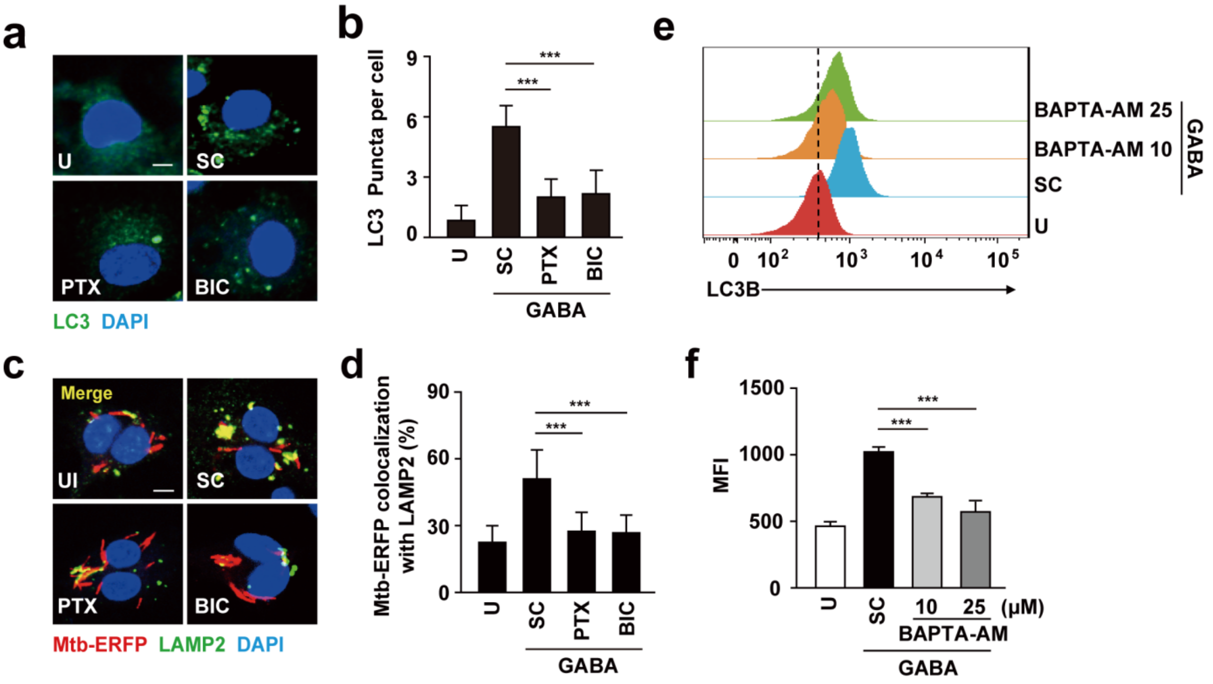

Supplementary Figure 13. Intracellular calcium release and GABA$_A$R-dependent signaling contribute to GABA-mediated autophagy activation in macrophages. (**a** and **b**) BMDMs were pretreated with bicuculline (BIC; 100 μM) or picrotoxin (PTX; 100 μM) for 1 h, and then treated with GABA (100 μM) for 18 h. Cells were stained with LC3 (green) and DAPI (for nuclei; blue). (**a**) Cells were visualized by confocal microscopy. Scale bars, 5 μm. (**b**) Quantitative data of LC3 punctate analysis. (**c** and **d**) BMDMs were preincubated with PTX (100 μM) or BIC (100 μM) for 1 h, infected with Mtb-ERFP (MOI of 5) for 4 h, and then treated with GABA (100 μM) for 18 h. Cells were stained with LAMP2 (green) and DAPI (blue). (**c**) Cells were visualized by confocal microscopy. Scale bars, 5 μm. (**d**) Quantitative data of colocalization of Mtb-ERFP and LAMP2 per cell. (**e** and **f**) BMDMs were pretreated with BAPTA-AM (10 or 25 μM) for 1 h and then incubated with GABA (100 μM) for 18 h. (**e**) The expression of LC3B was analyzed by flow cytometry. Representative gating strategy is shown in Supplementary Fig. 5. (**f**) Average MFIs of LC3B**.** ***$p < 0.001$. U, untreated; SC, solvent control (0.05% EtOH, for **a**-**d**; 0.1% dimethyl sulfoxide, for **e**,**f**). Statistical significance was determined by one-way ANOVA (**b**,**d**,**f**). Data shown (means ± SEM) represent

combined results of duplicate from two experiments (**b**,**d**,**f**). Images are representative of three independent experiments (**a,c**,**e**).

Supplementary Figure 14

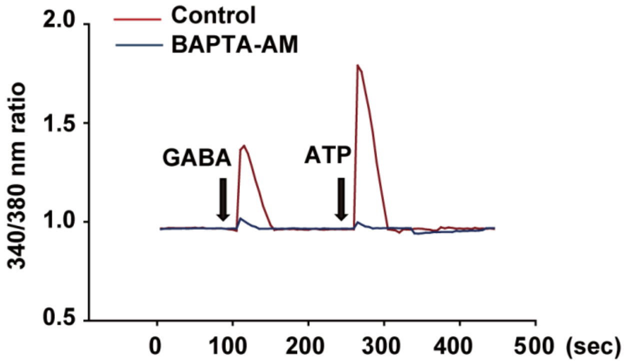

Supplementary Figure 14. Effect of BAPTA-AM on intracellular calcium response in BMDMs. BMDMs were pretreated with or without BAPTA-AM (25 μM) for 1 h. Cells were loaded with Fura-2 AM and incubated with GABA (100 μM). Data are representative of three independent experiments.

Supplementary Figure 15

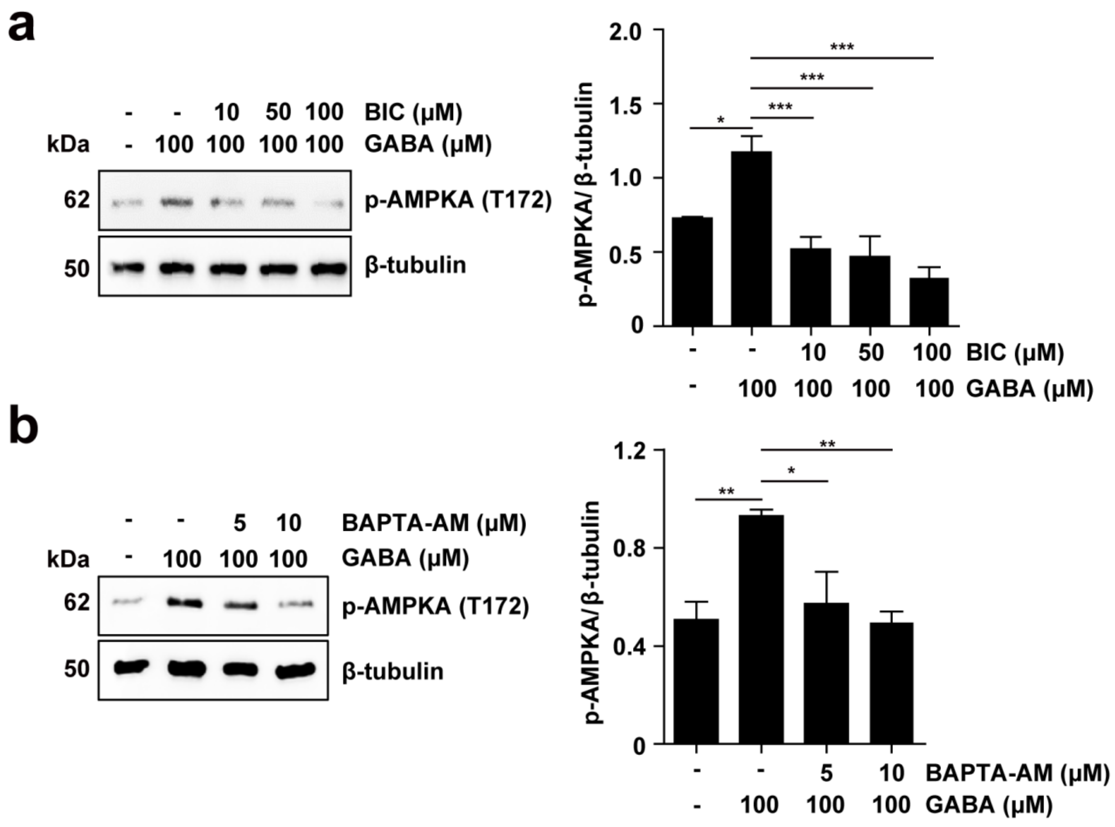

Supplementary Figure 15. $GABA_AR$ blockade inhibits GABA-induced AMPK activation. BMDMs were pretreated with bicuculline (BIC; **a**) or BAPTA-AM (**b**) or for 1 h, and then treated with GABA for 30 min. Representative immunoblots for the expression of the indicated proteins (left). The densitometric values for p-AMPKA were normalized to β-tubulin (right). *p < 0.05, **p < 0.01, and ***p < 0.001. Statistical significance was determined by one-way ANOVA. Data shown (means ± SEM) represent combined results of duplicate from four independent experiments. Images are representative of four independent experiments.

Supplementary Figure 16

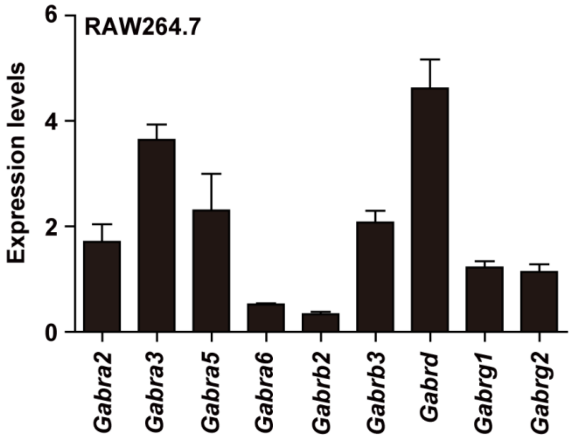

Supplementary Figure 16. Expression of $GABA_A$R subunits from RAW264.7 cells. In RAW264.7 cells, 9 $GABA_A$R subunit mRNAs were determined using the $\Delta\Delta$Ct method, and normalized to the reference gene *Gapdh*. Data shown represent combined results of triplicate from three independent experiments.

Supplementary Figure 17

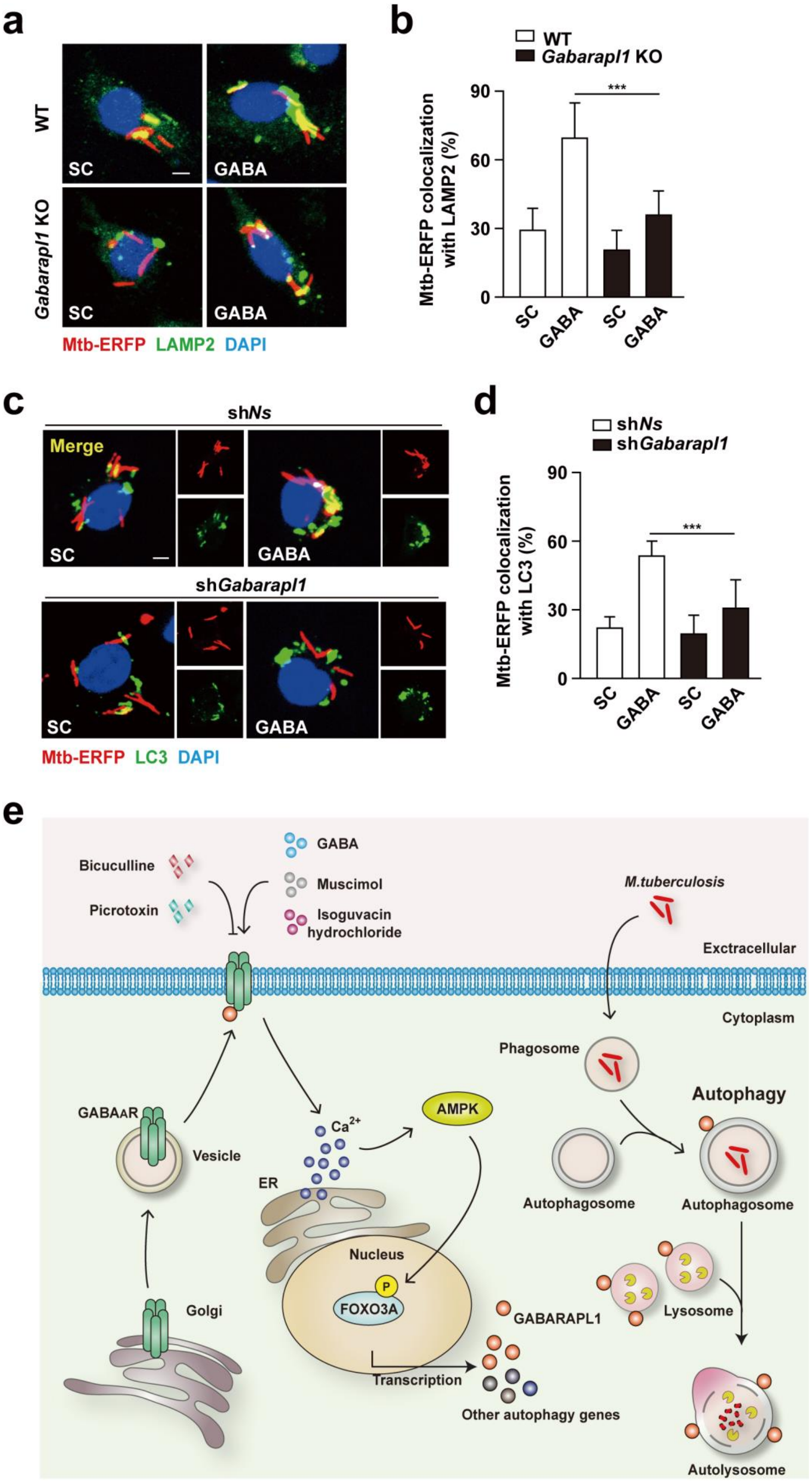

Supplementary Figure 17. GABARAPL1 is required for GABA-mediated phagosomal maturation against mycobacterial infection. (**a** and **b**) BMDMs from WT or *Gabarapl1* KO were infected with Mtb-ERFP (MOI of 5) and treated with GABA (100 μM). Cells were stained with LAMP2 (green) and DAPI (blue). (**a**) Cells were visualized by confocal microscopy. Scale bars, 5 μm. (**b**) Quantitative data of colocalization of Mtb-ERFP and LAMP2 per cell. (**c** and **d**) BMDMs were transduced with lentivirus expressing sh*Ns* or sh*Gabarapl1* using polybrene (8 μg/ml). After 36 h, BMDMs were infected with Mtb-ERFP (MOI of 5) for 4 h and incubated with GABA (100 μM) for 18 h. (**c**) Cells were visualized by confocal microscopy. Scale bars, 5 μm. (**d**) Quantitative data of colocalization of Mtb-ERFP and LC3 per cell. (**e**) Diagram representing the proposed mechanism. ***$p < 0.001$. ns, not significant; SC, solvent control (0.1% PBS). Statistical significance was determined by two-way ANOVA (**b**,**d**). Data shown (means ± SEM) represent combined results of duplicate from two experiments (**b**,**d**). Images are representative of three independent experiments (**a**,**c**).

Supplementary Figure 18

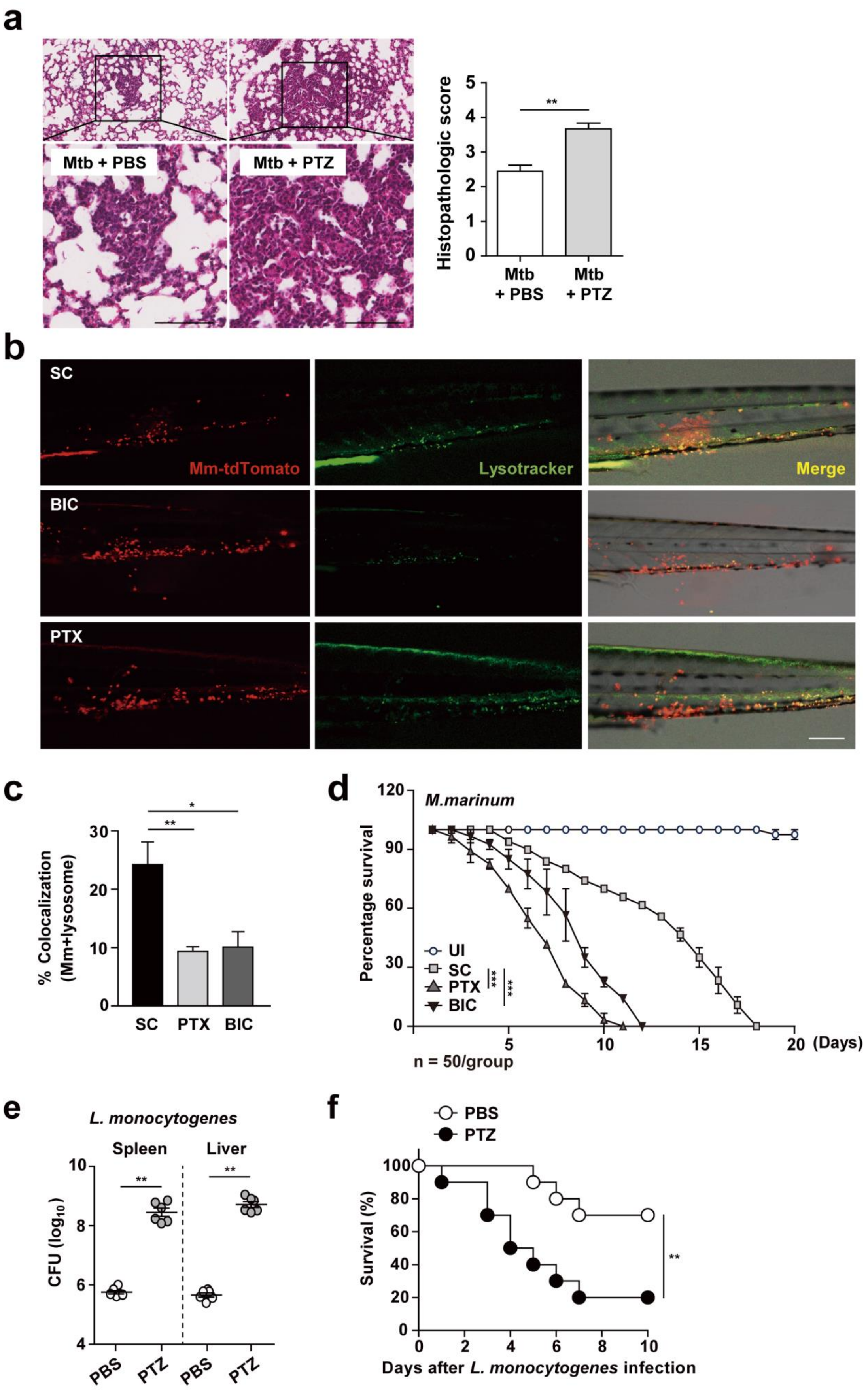

Supplementary Figure 18. GABAergic inhibition attenuates antimicrobial activities against intracellular bacterial infections *in vivo*. (**a**) Representative H&E-stained images were taken of lung tissue of mice as in Fig. **7a** (Mtb) (left). Scale bars, 100 μm. Quantitative analysis of histopathology scores was also performed (right). (**b** and **c**) Zebrafish embryos were infected with *M. marinum*-tdTomato (150 CFU) and then treated with bicuculline (BIC; 50 μM) or picrotoxin (PTX; 5 μM) as in Fig. **7e–f**. (**b**) Representative confocal images of embryos at 3 dpi. Zebrafish embryos were stained with Lysotracker (10 μM) for 1 h and then visualized by confocal microscopy. Scale bars, 1 μm. (**c**) Quantitative data of colocalization analysis of *M. marinum*-tdTomato and Lysotracker. (**d**) WT flies were injected with *M. marinum* (500 CFU) and then maintained with or without PTX (100 μM) or BIC (100 μM). Dead flies were counted in 24 h intervals (n = 50 per group). (**e** and **f**) Mice were injected i.p. with PBS or PTZ (40 mg/kg) 6 h before i.v. infection with *L. monocytogenes* (1×10^4 CFU). (**e**) Viable cell count of intracellular *L. monocytogenes* in spleen and liver of mice (n = 6 per group) at 5 dpi. (**f**) Survival of mice (n = 10 per group). *p < 0.05, **p < 0.01, and ***p < 0.001. SC; solvent control (0.01% EtOH, for **c**,**d**). Statistical significance was determined by Mann-Whitney U test (**a** right,**e**), one-way ANOVA (**c**), and log-rank (Mantel-Cox) test (**d**,**f**). Data (means ± SEM) are pooled from two independent experiments (**c,e**). Data are representative of three or four independent experiments. Each symbols represents one animal (**e**)

Supplementary Figure 19

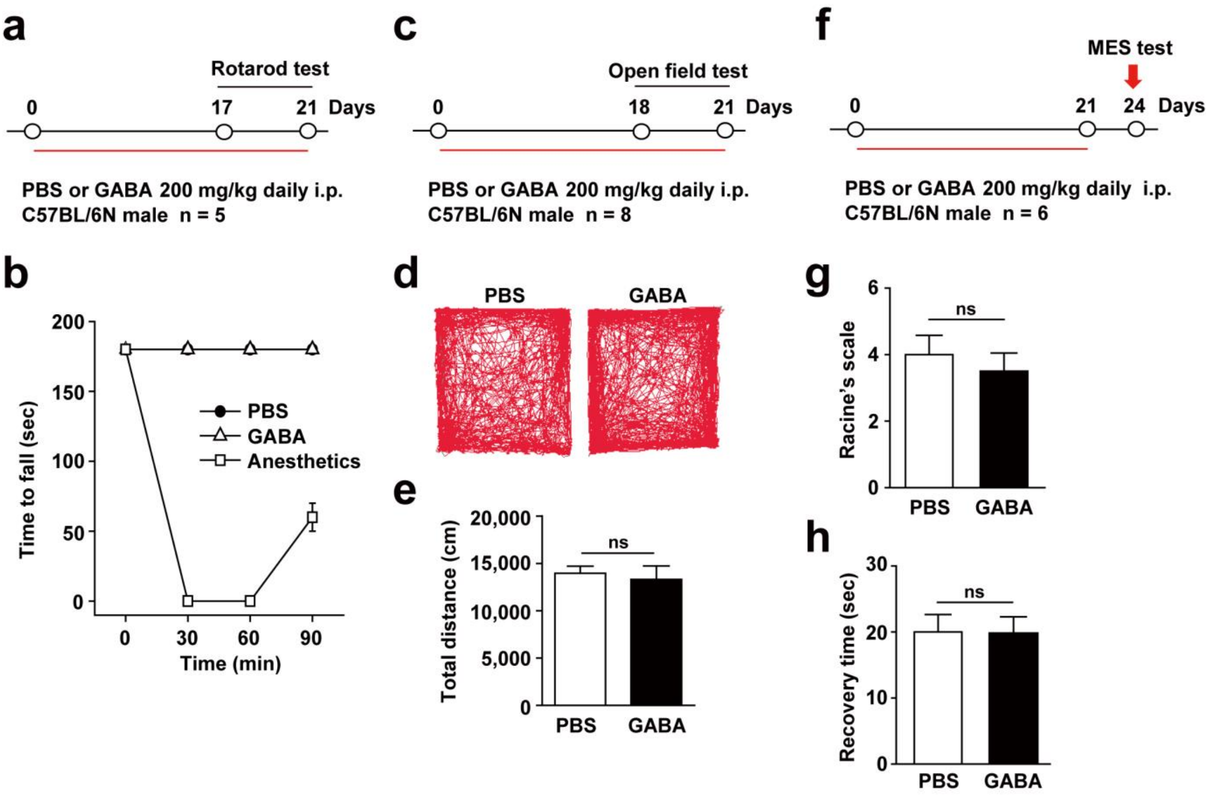

Supplementary Figure 19. Behavior tests for GABA-treated mice. (**a**) Schematic illustration of the experimental timeline for GABA injection and Rotarod test. (**b**) Mice treated with PBS (*n* = 5) or GABA (*n* = 5) were subjected to measure the motor performance. Results are shown as the maximum possible running time on the Rotarod (6 rpm, max time 180s) with error bars indicating S.E.M. (**c**) Schematic illustration of the experimental timeline for GABA injection and open field test. (**d** and **e**) Effects of GABA on horizontal locomotor activity was measured using the open field test. Representative traces of mouse movement during the open field test (60 min) are shown in (**d**). Results shown as the total distance of mouse movement are summarized in PBS (*n* = 8) and GABA (*n* = 8) treated groups (**e**). (**f**) Schematic illustration of experimental timeline for GABA injection and electroshock (ES) seizure test. (**g** and **h**) Effects of GABA on seizure susceptibility were evaluated using ES (6 pA, 0.25sec)-induced seizure. ES were applied by ear clip electrodes in mice treated with PBS (*n* = 5) or GABA (*n* = 5). The ES-induced seizure severity estimated by Racine`s scale

(**d**) and the seizure lasting time (**e**) were summarized. ns, not significant.

Supplementary Figure 20

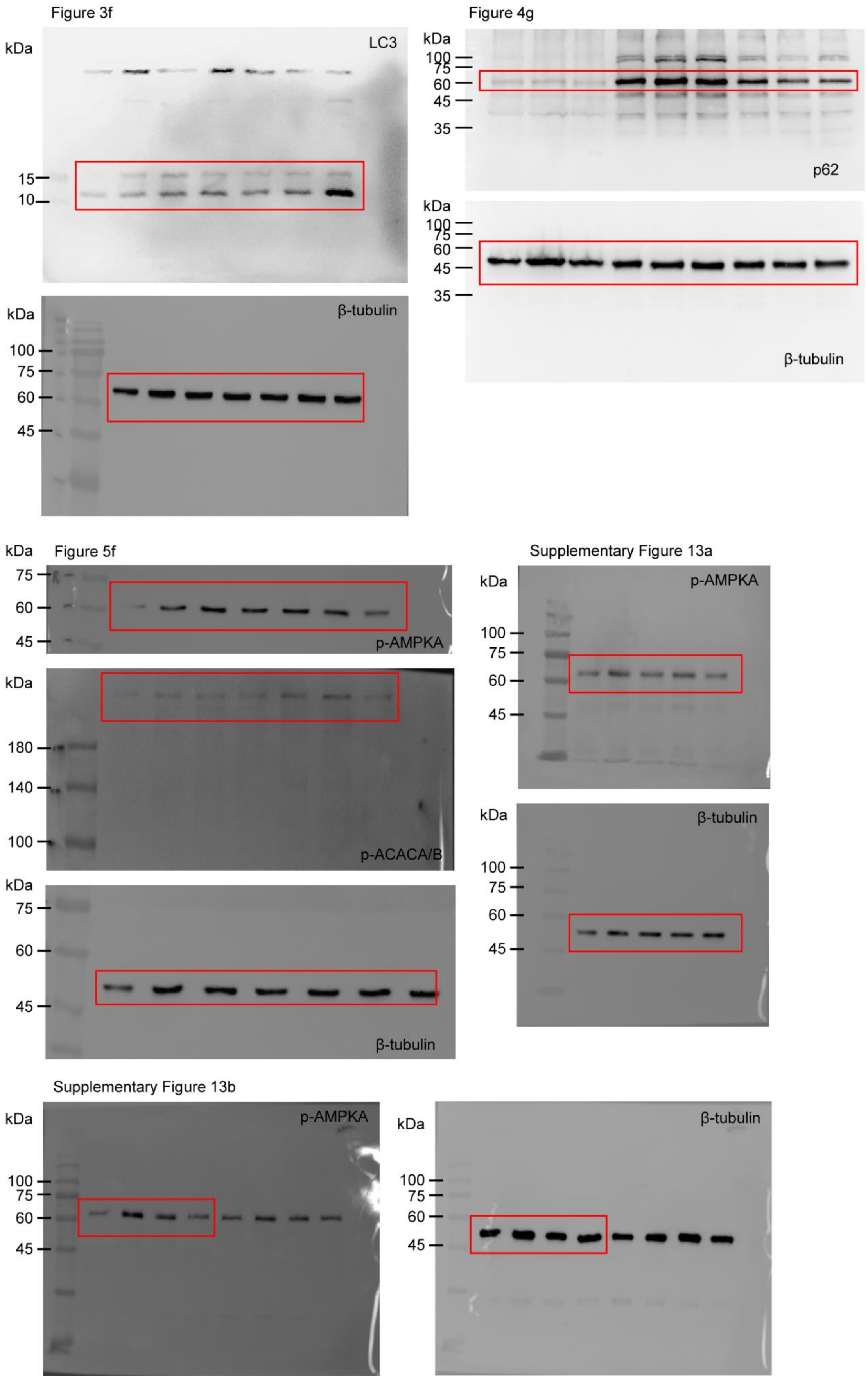

Supplementary Figure 20. Uncropped images of western blot. Red boxes show the region cropped from each blot and presented. Each figure corresponds to the western blots in the indicated figure number.

Supplementary Table 1. Primers used in this study.

Genes	Forward (5' → 3')	Reverse (5' → 3')
m*Gabarap*	AAGAGGAGCATCCGTTCGAGA	GCTTTGGGGGCTTTTTCCAC
m*Gabarapl1*	CATCGTGGAGAAGGCTCCTA	ATACAGCTGGCCCATGGTAG
m*Gabarapl2*	TCGGGCTCTCAGATTGTTGAC	ATGGCCTTCTCGGAGGGAA
m*Ambra1*	TCCAGAGAGCACCCAATTTACC	CTGTCACCATTGTCCTCAAAGT
m*Atg4c*	AGATGAAAGCAAGATGTTGCCT	CCCTGTAGGTCAGCCATATTCTA
m*Becn1*	GGAAAAGAACCGCAAGGTGGTG	AAACTGTCCGCTGTGCCAGATG
m*Map1lc3b*	CCCACCAAGATCCCAGTGAT	CCAGGAACTTGGTCTTGTCCA
m*Lamp1*	CTCTGCCTCCTTTCTGACCA	GCAGGGAAATGTTCACGAT
m*Atg16l2*	GCAGCTTGTGCAGCGTAAG	CTGGTTGGCCCTCTCTCTAC
m*Atg3*	ACACGGTGAAGGGAAAGGC	TGGTGGACTAAGTGATCTCCAG
m*Atg7*	TGCCTATGATGATCTGTGTC	CACCAACTGTTATCTTTGTCC
m*Tnf*	AGCACAGAAAGCATGATCCG	CTGATGAGAGGGAGGCCATT
m*Il6*	ACAAAGCCAGAGTCCTTCAGA	TGGTCCTTAGCCACTCCTTC
mactb	CCACCATGTACCCAGGCATT	AGGGTGTAAAACGCAGCTCA
m*Gapdh*	TGGCAAAGTGGAGATTGTTGCC	AAGATGGTGATGGGCTTCCCG
m*Gabra2*	GCTACGCTTACACAACCTCAGA	GACTGGCCCAGCAAATCATACT
m*Gabra3*	GCCGTCTGTTATGCCTTTGTATTT	TTCTTCATCTCCAGGGCCTCT
m*Gabra4*	AGAACTCAAAGGACGAGAAATTGT	TTCACTTCTGTAACAGGACCCC
m*Gabra5*	GATTGTGTTCCCCATCTTGTTTGGC	TTACTTTGGAGAGGTGGCCCCTTTT
m*Gabra6*	GGTGACCGGGCATCCCAGTGA	TGTTACAGCACCCCCAAATCCTGGC
m*Gabrb2*	GCTGGTGAGGAAATCTCGGTCCC	CATGCGCACGGCGTACCAAA
m*Gabrb3*	GAGCGTAAACGACCCCGGGAA	GGGACCCCCGAAGTCGGGTCT
m*Gabrg1*	ATCCACTCTCATTCCCATGAACAGC	ACAGAAAAAGCTAGTACAGTCTTTGC
m*Gabrg2*	ACTTCTGGTGACTATGTGGTGAT	GGCAGGAACAGCATCCTTATTG
m*Gabrd*	TCAAATCGGCTGGCCAGTTCCC	GCACGGCTGCCTGGCTAATCC
m*Gabrq*	GCTGGAGGTGGAGAGCTATGGCT	CCCCAGGTACGTGTACTGAGGGA
h*GABRA1*	GGATTGGGAGAGCGTGTAACC	TGAAACGGGTCCGAAACTG
h*GABRB1*	CCAGGTCGACGCCCACGGTA	GTGGCCTTGGGGTCGCTCAC
h*GABRR2*	CCTAGAAGAGGGCATAGACATCG	TCCAGTAGCTGCTGCATTGTTTG
h*GAPDH*	TCTCCTGCGACTTCAACA	TGGTCCAGGGTTTCTTACT

Supplementary Methods

Antibodies and reagents

Anti-GAD67 (PA5-21397) was purchased from Thermo Fisher Scientific. Lysotracker Green DND-26 (L7526) was purchased from Invitrogen. Anti-rabbit IgG isotype control (1:100 diluted; sc-2027) and F4/80 (1:100 diluted; sc-52664) were purchased from Santa Cruz. ABCA3 (1:100 diluted; ab24751) was purchased from Abcam. Ly6G (1:100 diluted; BP0075-1) was purchased from BioXcell. 4,5,6,7-tetrahydroisothiazolo-[5,4-c]pyridin-3-ol (THIP; T101) was purchased from Sigma Aldrich. Anti-mouse CD11b (1:100 diluted; 11-0112-81) and anti-mouse F4/80 (1:100 diluted; 17-4801-82) were purchased from Thermo Fisher Scientific.

***M. marinum* infection and intracellular bacterial growth in *D. melanogaster*.** Flies were maintained on standard cornmeal medium at 25 °C and 80% humidity. For *M. marinum* infections, 3–5-day-old male W^{1118} flies were anesthetized with CO_2 and injected with 500 CFU *M. marinum* in 50 nl (total volume) of PBS using an individually calibrated pulled-glass needle attached to a microinjector (IM-5B; Narishige, Japan). Then, *M. marinum*-infected flies were incubated and maintained at 25 °C and 60% humidity. All flies were transferred to fresh vials at least twice a week. To determine bacterial growth, *M. marinum*-infected live W^{1118} flies were incubated with various media (PTX or BIC) for the indicated days (n = 50/group, three independent experiments).

***Listeria monocytogenes* culture and mouse infection.** *L. monocytogenes* was kindly provided by Dr. C. S. Yang (Hanyang University, Korea) and grown in brain heart infusion (BHI; LPS solutions) broth at 37 °C for 16 h. A 1:10 dilution of the bacterial culture was grown for 2 h at 37°C prior to infection. Bacterial numbers were counted on a BHI agar plate. Mice were injected intravenously with *L. monocytogenes* (1×10^4 CFU/mouse). For

measurement of the bacterial burdens in liver and spleen, mice were killed on day 5 after inoculation, organs were homogenized in PBST, and serial dilutions of the homogenates were plated on BHI agar plates, with colonies counted 24 h later.

Macrophage depletion. Liposomes loaded with PBS or clodronate (Encapsula nanosciences) were prepared as described[1]. Mice were injected i.v. (200 μl) or i.n. (100 μl) with clodranate liposomes 24 h before infection. All clodronate liposome treatments have previously been shown to deplete macrophages within 24 - 48 h[2-5].

RNA extraction and RT-PCR analysis. Total RNA from homogenized lung was isolated using TRIzol reagent (Thermo Fisher Scientific, 15596-026), in accordance with the manufacturer’s instructions. After RNA quantitation, cDNA was synthesized by reverse transcription using the reverse transcriptase premix (Elpis Biotech, EBT-1515). For real-time RT-PCR analysis, real-time PCR was carried out using cDNA, primers, and SYBR Green master mix (Qiagen, 204074). Reactions were run on a Rotor-Gene Q 2plex system (Qiagen, Germany). To analyze real-time PCR data, we performed relative quantification using the $2^{\Delta\Delta}$ threshold cycle (Ct) method using *Actb* for normalization. Data are expressed as relative fold changes. The primer sequences used in this paper were shown in Supplementary Table 1.

ELISA analysis. For the measurement of TNF-α or IL-6 production in BMDMs, ELISA was performed. Supernatants were analyzed using a Mouse ELISA Kit to detect TNF-α (BD OptEIA™, Mouse TNF ELISA Set II) and IL-6 (BD OptEIA™, Mouse IL-6 ELISA Set). All assays were performed as recommended by the manufacturer.

Rotarod test. Motor performance in mice treated with PBS (*n* = 5) or GABA (*n* = 5) was evaluated by rotarod analysis as described previously[6]. To administer the rotarod test, mice were subjected to a training period of 5 min on an accelerating rotarod (SciTech Korea Inc., Seoul, Korea) three times a day for four consecutive days. Mice were placed on a cylindrical platform (wide 12 cm, diameter 6 cm) suspended 33 cm above the bottom of the apparatus. Measurements were recorded on the fifth day with at a setting of 6 r.p.m. Duration of time on the rotating rod was recorded. at each time point, and the cut-off time for this test was 3 min. Mice of the positive control group received the combination of Zoletil (1.2 mg/kg) and xylazine HCl (0.24 mg/kg).

Open field test. Activity was tested and compared in mice treated with PBS (*n* = 5) or GABA (*n* = 5) as described previously[7]. The open field was a dimly lit 40×40×40 cm box in which the mice were observed and tracked using EthoVision XT 11.5 software (Noldus, Wageningen, The Netherlands) for 60 min each on three consecutive days.

Seizure susceptibility. To induce electroshock seizures, the same electric stimulus (6 mA, 50Hz, 0.2 sec) was applied through a pair of ear clip for all animals, produced by a Rodent Shocker 221 (Harvard Apparatus, MA, U.S.A.). Mice were challenged with the median current strength value (CS_{50}) predicted to produce tonic hindlimb extension in 50 % of the animals tested[8]. The electroconvulsive response was evaluated by Racine`s scale, and monitored until animals recovered to normal behavior to compare in PBS- (*n* = 5) and GABA-injected mice (*n* = 5).

Supplementary References

1. Van Rooijen, N. & Sanders, A. Liposome mediated depletion of macrophages: mechanism of action, preparation of liposomes and applications. *J. Immunol. Methods* **174**, 83-93 (1994).

2. Breitbach, K., Klocke, S., Tschemig, T., van Rooijen, N., Baumann, U. & Steinmetz, I. Role of inducible nitric oxide synthase and NAPDH oxidase in early control of *Burkholderia pseudomallei* infection in mice. *Infect. Immun.* **74**, 6300-6309 (2006).

3. Cote, C.K., Van Rooijen, N. & Welkos, S.L. Roles of macrophages and neutrophils in the early host response to Bacillus anthracis spores in a mouse model of infection. *Infect. Immun.* **74**, 469-480 (2006).

4. Ramos, P. et al. Macrophages support pathological erythropoiesis in polycythemia vera and β-thalassemia. *Nat. Med.* **19**, 437-445 (2013).

5. Sun, K. & Metzger, D.W. Inhibition of pulmonary antibacterial defense by interferon-gamma during recovery from influenza infection. *Nat. Med.* **14**, 558-564 (2008).

6. Choi, K.H. et al. Alpha-lipoic acid treatment is neurorestorative and promotes functional recovery after stroke in rats. *Mol. Brain* **8**, 9 (2015).

7. Jang, Y. et al. A high-fat diet induces a loss of midbrain dopaminergic neuronal function that underlies motor abnormalities. *Exp. Neurobiol.* **26**, 104-112 (2017).

8. Giardina, W.J. & Gasior, M. Acute seizure tests in epilepsy research: electroshock- and chemical-induced convulsions in the mouse. *Curr. Protoc. Pharmacol.* **Chapter 5**:Unit 5.22. (2009).
